# Nanomaterials in Cosmetics: Recent Updates

**DOI:** 10.3390/nano10050979

**Published:** 2020-05-20

**Authors:** Georgios Fytianos, Abbas Rahdar, George Z. Kyzas

**Affiliations:** 1 Department of Chemistry, International Hellenic University, 65404 Kavala, Greece; 2 Department of Food Science and Technology, International Hellenic University, 57400 Sindos, Greece; 3 Department of Physics, Faculty of science, University of Zabol, Zabol 538-98615, Iran; a.rahdar@uoz.ac.ir

**Keywords:** nanomaterials, cosmetics, regulations, toxicity, safety

## Abstract

This review paper collects the recent updates regarding the use of nanomaterials in cosmetics. Special focus is given to the applications of nanomaterials in the cosmetic industry, their unique features, as well as the advantages of nanoscale ingredients compared to non-nanoscale products. The state-of-the-art practices for physicochemical and toxicological characterization of nanomaterials are also reviewed. Moreover, special focus is given to the current regulations and safety assessments that are currently in place regarding the use of nanomaterials in cosmetics—the new 2019 European guidance for the safety assessment of nanomaterials in cosmetics, together with the new proposed methodologies for the toxicity evaluation of nanomaterials. Concerns over health risks have limited the further incorporation of nanomaterials in cosmetics, and since new nanomaterials may be used in the future by the cosmetic industry, a detailed characterization and risk assessment are needed to fulfill the standard safety requirements.

## 1. Introduction

Nanomaterials belong to a large “section” of materials, and their use has garnered great attention due to their significant physicochemical properties [1]. Among the first industries to implement nanotechnology-based materials is the cosmetics industry [2]. Nano-based ingredients have been in use in the cosmetic industry for more than 30 years [3,4,5]. In the EU, the official definition of a nanomaterial in cosmetics is given as: “an insoluble or bio-persistent and intentionally manufactured material with one or more external dimensions, or an internal structure, on the scale from 1 to 100 nm” [6].

**Figure 1:**
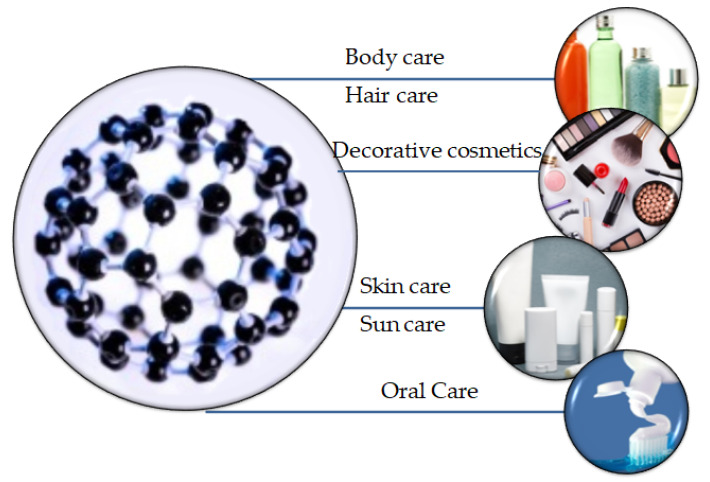
Nanomaterials in various cosmetic applications.

Nanomaterials-based cosmetics show some unique advantages compared to micro-scale cosmetics. The use of nanomaterials (NMs) by the cosmetic industry aims for long-lasting effects and increased stability. The high surface area of nanomaterials allows for more efficient transport of the ingredients through the skin [7]. Some of the main targets of using nanomaterials in cosmetics could be the efficient penetration into the skin for the improved delivery of the ingredients of the product, new color elements (e.g., in lipsticks, and nail polishes), transparency (e.g., in sunscreens), and long-lasting effects (e.g., in makeup) (Figure 1). The ultimate goal of the cosmetic industries when using NMs is to deliver the right amount of ingredients to the desired parts of the body, and to attain long term stability. Currently, the most common use of NMs in cosmetics is in skincare products, and particularly, in sunscreens to act as UV filters.

In 1986, Christian Dior launched the anti-aging cream Capture^TM^ which was based on liposomes. Over the years, hundreds of cosmetic products began to use NMs, and various world-famous cosmetic brands use NMs in their products [8]. L’Oréal S.A., which invests a great amount of revenue in nanotechnology, ranks sixth in the United States in the number of obtained nanotechnology-related patents [9], and uses up to four nano-ingredients (i.e., TiO_2_, ZnO, silica, carbon black) in some of their formulas [10]. Shiseido uses nano-TiO_2_ and ZnO in wet-based formulas (e.g., emulsions), but does not use them in aerosols due to the risk of inhaling [11]. In general, the world-famous cosmetic industries are gradually incorporating nanomaterials in their products [12].

The World Health Organization (WHO), non-governmental organizations, political institutions and agencies have raised concerns about the safety of NMs and their use in consumer goods [3]. Currently, the European Commission (EC) has updated the guidance on the safety assessment of NMs in cosmetics [13], and the Food and Drug Administration (FDA) has developed its own guidance for the application of nanotechnology in industrial products [14]. In Europe, future toxicological data for hazard identification must not include any animal testing, since it is strictly prohibited under the EC Cosmetic Regulation No 1223/2009, and alternative methods, e.g., ex vivo or in vitro, must be used for safety evaluation.

In the beginning of 2020, The European Union Observatory for Nanomaterials (EUON) [15], announced that all companies that manufacture, use, or import nanoforms should have a REACH registration compliant (REACH is a regulation of the European Union, and stands for Registration, Evaluation, Authorization, and Restriction of Chemicals). The purpose of this is for companies to offer end users adequate information regarding the safety of the products. This applies to all registrations of all nanoforms within the scope of REACH. NMs that fall within the scope of REACH but are not registered are considered illegal. Based on the recent catalogue of NMs, the EC asked the Scientific Committee on Consumer Safety (SCCS) for a priority list of nanomaterials used in cosmetics for the purpose of risk assessment.

In this review, the nanomaterials safety assessment process is presented, followed by the current regulation status in Europe and the USA. The types of nanomaterials that are currently used in cosmetics are also discussed, followed by the recent status and advances regarding production and characterization of nanomaterials.

## 2. Types of Nanomaterials in Cosmetics

### 2.1. Inorganic Nanoparticles

Inorganic nanoparticles are non-toxic, hydrophilic, biocompatible, and highly stable compared to organic nanoparticles. Their major difference—apart from the aforementioned—is that inorganic nanoparticles are synthesized from inorganic elements (Ag, Au, Ti, etc.), while the organic ones are synthesized from polymers. One of the most widely used inorganic nanoparticles for sunscreens is TiO_2_, and in nanoscale it has a higher sun protection factor (SPF) which makes it more efficient, and has a better cosmetic result due to its transparency, compared to TiO_2_ pigment. Oftentimes, in the market, companies use words such as “sheer” or “invisible” when nanoscale TiO_2_ or ZnO are used. It is reported that nanoscale TiO_2_ and ZnO show great advantages over many products at larger than nano-dimensions [16]. Micro-TiO_2_ and ZnO are used as ingredients in sunscreens due to their UVA and UVB absorption capabilities. Nanoparticles of ZnO and TiO_2_ are also widely used in sunscreens as UV filters [8] starting at the size of 20 nm. They show better dispersion and leave a better cosmetic result. For the analysis of ZnO and TiO_2_ in cosmetic products, the combination of Transmission Electron Microscopy (TEM) and X-ray Powder Diffraction (XRD) is considered an efficient approach [17]. Regarding their safety, inhalation of high concentrations of ZnO nanoparticles has been reported to cause health damage [18]. However, a different route of exposure (i.e., the dermal route), for ZnO concentrations in typical sunscreen formulas, are considered safe since there is neither proof for penetration into the viable epidermis nor toxicity issues [19].

### 2.2. Silica (SiO_2_)

Silica nanoparticles have attracted interest from the cosmetic industry, because they show hydrophilic surface favoring protracted circulation and thanks to their low production cost [20]. Nano Silica is used to enhance the effectiveness, texture, and shelf-life of cosmetic products. It adds absorbency and acts as an anti-caking agent [21]. It has been indicated that silica nanoparticles may help improve the appearance and distribution of pigments in lipsticks, and prevent pigments from migrating into the fine line of lips [22]. Silica nanoparticles are stabilized nanodispersions with a range size of 5 to 100 nm, which can deliver lipophilic and hydrophilic substances to their site of action by encapsulation [23]. Silica nanoparticles can be found in leave-on and rinse-off cosmetic products for hair, skin, lips, face and nails, and an increase of silica nanoparticles presence in cosmetic products is anticipated [24]. Results are controversial regarding the safety of silica-based nanoparticles and factors such as size and surface modifications should be taken into account when assessing toxicity [24,25,26,27]. Therefore, opinions regarding the use and exposure of silica nanoparticles in cosmetics are still inconclusive, and further long-exposure tests are needed.

### 2.3. Carbon Black (Nano)

Carbon Black, CI 77266, is a known cosmetic ingredient that is often used as a colorant for eye decorative cosmetic products, skin products, and mascaras. Currently, in its nano form, it is authorized in the EU, and it is being used as colorant at a maximum concentration of 10%. Research on carbon black nanoparticles showed that they exhibited a higher propensity of inducing cytotoxicity, inflammation, and altered phagocytosis in human monocytes than the micron size nanoparticle [28]. When there is no risk of inhalation, it is considered safe to be used in cosmetic products in the EU [29].

### 2.4. Nano-Organic Materials

Tris-Biphenyl Triazine is a very effective and very photostable filter, making it a unique choice ingredient for the formulation of sunscreens [30]. Tris-Biphenyl Triazine (nano) works as a broad-spectrum UV filter, suitable for sunscreen products and anti-aging face care products. It presents significant photostability and is an authorized UV filter in Europe. BASF SE is using it under the name TINOSORB^®^ A2B [31]. Methylene bis-benzotriazolyl tetramethylbutylphenol (nano) or MBBT, is an authorized UV filter in the EU market, and can be used at concentrations of up to 10% w/w for dermally applied cosmetic products. Based on SCCS’ opinion [32], MBBT does not pose a risk to humans if applied on healthy, intact skin. However, SCCS expressed concerns regarding possible irritation effects and potential bioaccumulation in selected tissues.

### 2.5. Nano-Hydroxyapatite

Nano-hydroxyapatite has been used in oral care and cosmetic products, being incorporated in various products for the treatment of dental hypersensitivity and enamel remineralization [33] and is considered to be a promising and safe option for oral care products [34]. Nano-hydroxyapatite particles have been integrated into oral care products, such as dentifrices and mouthwashes, and due to its remineralization and desensitization properties, nano-hydroxyapatite could be an alternative to fluoride toothpaste [35].

### 2.6. Gold and Silver Nanoparticles

**Figure 2:**
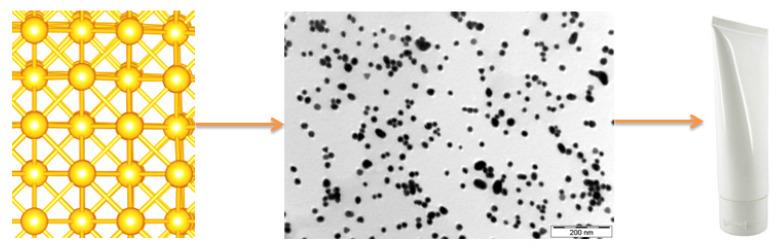
Gold nanoparticles use in cosmetic products.

Gold [36,37,38,39,40,41,42,43,44,45,46] and silver nanoparticles [47,48,49,50,51,52], apart from the numerous applications that they have, also show antibacterial and antifungal properties [53]. Gold and silver nanoparticles are used in cosmetic products (Figure 2) such as deodorants and anti-aging creams. In Europe, the SCCS, due to several major data gaps, has yet to draw any conclusions regarding the safety of colloidal silver in nano form when used in oral and dermal cosmetic products [54]. In the USA, cosmetic products cannot claim antibacterial properties because that claim is based on a physiological function, and therefore it can only be used in drug products and not in cosmetics [55]. It has been reported that silver nanoparticles can be used as effective growth inhibitors in numerous microorganisms [56]. Silver and silver-based compounds are used to control bacterial growth in various applications [57,58,59]. The use of silver in cosmetics can be compromised since silver-based compounds gradually precipitate in solutions and emulsions, and a solution to this could be the use of silver nanoparticles. Kokura et al. [59] studied the use of silver nanoparticles as a preservative in cosmetics, and reported that silver nanoparticles remained stable, without exhibiting sedimentation, for longer than 1 year. In addition, silver nanoparticles showed acceptable preservation efficacy against bacteria and fungi, and did not penetrate human skin [59]. Pulit-Prociak et al. [60] studied the application of gold and silver nanoparticles in cosmetic formulations. They reported embedding differences between silver and gold nanoparticles into the structure of a cream. Silver nanoparticles introduced to the cream mixture agglomerate, but gold nanoparticles did not agglomerate after introduction to cream mixtures. They attributed this phenomenon to the greater value of the electrokinetic potential located on the surface of gold nanoparticles. Based on a model dermal membrane study, they have reported concerns over the penetration of nanoparticles into the skin for samples with nanoparticles concentration of 110–200 mg/kg. Due to the complex composition of cosmetic creams, it is not easy to characterize the primary gold nanomaterials in situ [61]. Cao et al. [61], developed a practical protocol including separation, quantification, and characterization of gold nanomaterials present in commercially available cosmetic creams.

### 2.7. Nanoliposomes

Liposomes at nanoscale are called nanoliposomes. They are concentric bilayered vesicles in which the aqueous volume is enclosed by a lipid bilayer of phospholipids [8]. Nanoliposomes are biodegradable and biocompatible, representing a highly adaptable ingredient category for the cosmetic field [9]. They are used as protective carriers of active ingredients (e.g., vitamins), for increasing skin permeability and for moisturizing purposes. They can be used for fragrance delivery in antiperspirants, body-spray deodorants and lipsticks [23]. Despite their promising features, low drug loading, low reproducibility, and physicochemical instability issues have limited their commercial applications in cosmetics [62,63].

### 2.8. Solid Lipid Nanoparticles (SLN) and Nanostructured Lipid Carriers (NLC)

SLNs are distinguishable from NLCs by the composition of their solid particle matrix and are an alternative carrier system to liposomes and emulsions [64]. They act as a carrier for ingredients due to their various advantages over existing conventional formulation, and they are excellent for skin hydration [65]. SLNs are composed of a single layer of shells, and the core is oily or lipoidal in nature [66]. The small size of SLNs ensures close contact with the stratum corneum, which increases the penetration of ingredients through the skin [64]. They can help to increase the water content on the skin and can also act as potential UV blockers. NLCs can carry more active ingredients but SLNs can improve chemical stability [55]. They exhibit easy scalability, in addition to their well-established production methods. They also show skin penetration improvement, biocompatibility and stability being an effective carrier delivery agent [23]. SLNs and NLCs can be found in moisturizing creams and sunscreens [66]. SLNs and NLCs are very well-tolerated carrier systems for dermal applications, and the production of these carrier systems is feasible in laboratory as well as on large scale [64].

### 2.9. Nanocapsules

Nanocapsules are polymeric NM capsules that are surrounded by an oily or water phase [67]. Nanocapsules are used in cosmetics for the protection of ingredients, for decreasing chemical odors and for resolving incompatibility issues between formulation components [67]. Polymeric nanocapsule suspensions can be directly applied on the skin as a final product, or incorporated into semisolid formulations as an ingredient. The degree of skin penetration of an ingredient can be modulated according to the polymer and the surfactant used as raw materials [68]. Stabilized poly-l-lactic acid nanocapsules with a diameter of approximately 115 nm were prepared through nanoprecipitation, and a sustained release of perfume, by entrapping fragrance molecules in a polymeric nano-carrier was achieved [69]. This kind of encapsulation of molecules in biocompatible nanocapsules can play a significant role in the future of deodorant products.

### 2.10. Dendrimers

Dendrimers possess a spherical architecture composed of a core from which symmetric units are built, and this structure is the main reason for the versatility of dendrimers [23]. Dendrimers are polymers, and due to their stability they help in delivering ingredients through the skin [55]. Dendrimers are used in shampoos, and deodorants [9]. The surface activity of dendrimers’ symmetrical branches is due to the hydrophobic properties of their edge part combined with the hydrophilic characteristics of the core [9]. Properties such as monodispersity, polyvalence, and stability make them ideal carriers for drug delivery [66]. Dendrimer structure has helped to increase the overall loading and skin penetration of resveratrol (known for its anti-oxidant and anti-aging properties) [70], which has led to the later scale-up and commercialization of this dendrimer structure-based product.

### 2.11. Nanoemulsions

Nanoemulsions are typically oil-in-water or water-in-oil colloidal dispersions, which contain droplets that range from a few nanometers to 200 nm in diameter [71]. An increase in patent-protection activities related to nanoemulsions shows growing industrial interest in it [72]. The small size of the droplets can provide the desirable optical, stability, rheological, and ingredient delivery properties, which are superior to conventional emulsions. Nanoemulsions tend to be transparent and stable. An oil-in-water (O/W) nanoemulsion containing *Opuntia ficus-indica* (L.) Mill hydroglycolic extract was produced, and was characterized by its thermal stability and moisturizing efficacy [73]. Results showed that O/W nanoemulsions containing 1% of *Opuntia ficus-indica* (L.) Mill extract presented suitable stability for at least 2 months. In addition, the formulation was able to increase the water content of stratum corneum, showing its moisturizing efficacy. Musazzi et al. [74] reported that nanoemulsions could significantly influence the permeation profiles of molecules as a function of their physicochemical properties, and in particular, O/W nanoemulsions significantly improved the permeation profiles of apolar ingredients in comparison to conventional emulsions.

### 2.12. Other Types

Nanoparticles in use in the cosmetic industry can be divided into two groups [75]: (i) biodegradable nanoparticles (e.g., liposomes and chitosan) and (ii) nonbiodegradable nanoparticles (e.g., polystyrene, ZnO, and silica-based nanoparticles). Chitin and its deacetylated derivative—chitosan—are of great interest to the cosmeceutical industry due to their unique biological and technological properties [76] A chitin nanofibril is an example of a nanocrystal obtained from the crustacean exoskeleton, eliminating carbonate and protein portions, while still being considered safe to use [75]. Chitin nanofibrils in emulsions can generate the formation of a hygroscopic molecular film that slows down water evaporation, and contributes to skin hydration [77].

## 3. Safety Considerations

Nanomaterials in cosmetics could have various functions (e.g., UVA and UVB filters in sunscreens, nano-preservatives). The unique characteristics of any given nanomaterial which may lead to the desired function/property of the cosmetic product may also pose a risk to the consumer. With this in mind, a standard safety evaluation of all nanomaterial is necessary, including tests dealing with the nano-characteristics (e.g., penetration into viable skin layers due to their small size as well as inhalation experiments in the case of sprays/powders). For FDA, some of the key points that should be included in the safety assessment according to the recent guidance [14] are the physicochemical characteristics, agglomeration, and size distribution of NMs, morphology, solubility, density, porosity, stability, and impurities. In addition, the potential exposure routes of NMs should be identified, and in vitro and in vivo toxicological data—including studies on dermal penetration and potential inhalation, genotoxicity studies, and possible skin and eye irritation studies—should be conducted.

**Figure 3:**
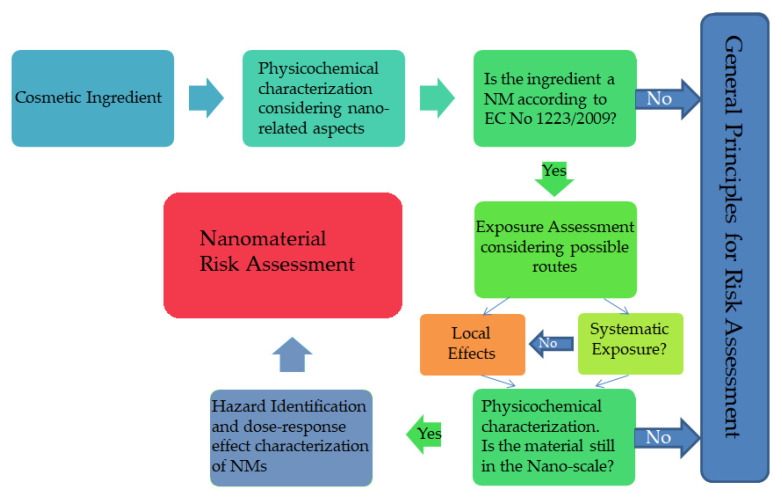
Schematic outline for the nanomaterial safety assessment in cosmetics based (but modified) on SCCS (Scientific Committee on Consumer Safety), Guidance on the Safety Assessment of Nanomaterials in Cosmetics, October 2019, SCCS/1611/19 [13].

The exposure assessment for NMs follows a similar procedure to non-NM ingredients, but with a special focus on the nano-aspects. In Figure 3, the schematic outline of the safety assessment of a cosmetic product containing NMs is presented. In Europe, there are NMs for which the SCCS has expressed inconclusive opinions, e.g., Colloidal Silver (nano) [54], Styrene/Acrylates copolymer (nano) and Sodium styrene/Acrylates copolymer (nano) [78], and Silica, Hydrated Silica, and Silica Surface Modified with Alkyl Silylates (nanoform), which is why the EC requested that the SCCS to should assess if a potential risk can be identified [79]. The inconclusiveness is due to the lack of data submitted by the Applicants.

**Figure 4:**
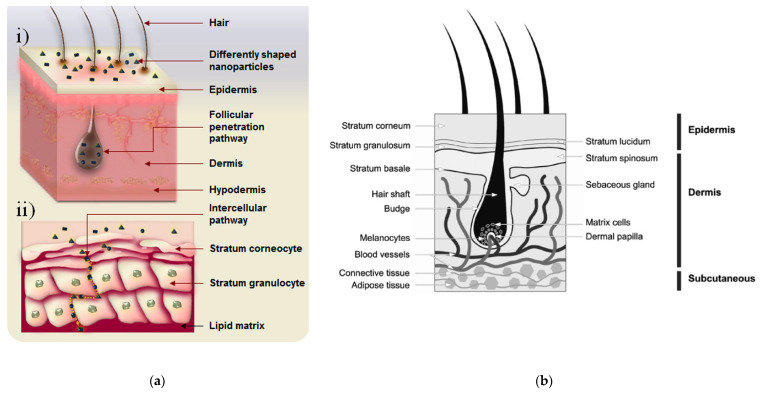
(**a**) Two main possible skin penetration pathways are illustrated. The nanomaterial (**i**) enters via hair follicles (the follicular penetration pathway) and (**ii**) diffuses through the gaps between corneocytes (the intercellular penetration pathway). Reproduced with permission from [80]. Copyright Springer Nature, 2015; (**b**) Schematic of the structure of human skin. The epidermis consists of five thin layers, with the outmost layer, the stratum corneum, largely providing the vital barrier function of the skin. Reproduced with permission from [2]. Copyright Elsevier, 2012.

One of the most important aspects to take into consideration is the NMs’ routes of exposure. The primary route is skin exposure with the stratum corneum as the first layer of epidermis. There are still some uncertainties regarding the possibility of NMs penetrating through the stratum corneum into viable layers, where toxicological concerns may arise [55]. In Figure 4, a schematic of the structure of the human skin is presented. Although very small NMs still have much larger molecular weights compared to known molecules which penetrate the skin, further tests for every NM to be used in a cosmetic formula should be performed. Extra attention to safety assessment should be given to sprays or aerosols that may contain NMs, because exposure via inhalation is possible. The SCCS Notes of Guidance (SCCS/1602/18) included a non-exhaustive list of parameters that are required for an exposure scenario. For NMs, in addition to the weight-based concentration of the NM, the concentration should also be given in terms of particle number concentration and surface area. Also, changes in the aggregation and/or degradation/dissolution status of the NM during exposure should be accounted for. Apart from skin exposure, oral exposure—to NMs existing in toothpastes, mouthwashes, and lipsticks—is also possible.

## 4. Regulations

In the EU, the main regulatory framework for cosmetic products is the EC Regulation 1223/2009. Based on this framework, a list of all NMs that are in use in cosmetics should be available to all consumers. The last updated catalogue consisted of 29 NMs. This catalogue is for consumer information, and it does not necessarily mean that all products listed in it are authorized NMs. Very recently (i.e., October 2019), the EC updated the existing guidance for nanomaterials in cosmetics with the Guidance on the Safety Assessment of Nanomaterials in Cosmetics, with the help of the SCCS (SCCS/1611/19). The SCCS provides guidance from industries to public authorities to ensure compliance with the EC No 1223/2009, regarding safety assessment of NMs intended for use as cosmetic ingredients. It should be noted that based on the Guidance SCCS/1484/12 from 2012, a complete animal testing ban has come in force under the Cosmetics Regulation since March 2013, and alternative methods for the safety assessment of cosmetic ingredients have been followed ever since (including NMs tests).

In Europe, safety assessment is mandatory for all ingredients of cosmetic products. The same applies to nanomaterials to be used in cosmetics. In the EU, all cosmetic products, prior to being placed on the market, must be electronically communicated to the EC through the Cosmetic Products Notification Portal (CPNP), by the Responsible Person (i.e., designated legal or natural person of a company) for the purposes of market surveillance and for prompt and appropriate medical treatment. The information provided includes the identification of the NMs present in the cosmetic products. By the end of 2018, less than 1.5% of cosmetic products notified in the CPNP were declared to contain nanomaterials [81]. It is mandatory to declare whether the cosmetic product includes any NMs, under the Regulation (EC) No 1223/2009. Even if the NMs to be used are not described in the Regulation (most nano-based colorants, UV filters and preservatives are included) a special procedure should be followed. In case of concerns, the EC can ask the SCCS for further investigation [6]. The EC should authorize the nano-ingredients prior to their use in a cosmetic product. When it comes to the labeling, all NMs should be listed in the cosmetic label starting with the name of the chemical followed by the word (nano) in brackets. In the latest updated catalogue for NMs [82] in cosmetics, it is clearly stated that the catalogue is “for information only and is expressly not a list of authorised nanomaterials”. Until now, the EC has authorized the use of the following NMs: UV filters containing (nano) TiO_2_, ZnO, MBBT, and tris-biphenyl triazine. It has also allowed carbon black (nano) for use as a colorant in cosmetic products.

The EC published a catalogue of all NMs that are used in cosmetic products in the EU in 2017, which was last updated on December 2019 (Table 1). It should be noted that many of the substances listed in Table 1 are also registered under the REACH Regulation.

The nanomaterials guidance (NG) focuses on physicochemical characterization and exposure assessment of the materials, together with data requirements for human health risk evaluation based on the ingredients. Safety assessment of nanomaterials has been updated with a focus on immunotoxicity, encapsulated nanomaterials and nano-carriers. Moreover, new methodological approaches are presented for the toxicity evaluation of nanomaterials. In the beginning of 2020, The European Union Observatory for Nanomaterials (EUON) [15], announced that all companies that manufacture, use, or import nanoforms should have a REACH registration compliant. The purpose is for companies to give to end users adequate information needed for the safety of the products. This goes for all registrations of all nanoforms in the scope of REACH. NMs that fall within the scope of REACH and are not registered are considered illegal. Based on the recent catalogue of NMs, the EC asked the SCCS for a priority list of NMs for risk assessment.

In the USA, the FDA monitors the use of NMs in cosmetics. In the FDA Nanotechnology Task Force Report of 2007 [83], the FDA presented an assessment of scientific and regulatory considerations relating to the safety and effectiveness of products containing NMs. The Task Force recommended the issue of a guidance describing safety issues for cosmetic products. Based on that, manufacturers should consider ensuring that cosmetic products made with NMs are safe. Still, there is no strict regulatory definition of nanotechnology and on NMs. In 2014, the FDA published a guidance for the industry titled “Final Guidance for Industry—Safety of Nanomaterials in Cosmetic Products” [14] which analyzes safety issues and provides guidance for cosmetic industries [55].

Finally, the International Cooperation on Cosmetics Regulation (ICCR), established in 2007 [84], is an international group of cosmetics regulatory authorities from Brazil, Canada, the EU, Japan, and the USA, which meets on an annual basis to address common issues on cosmetics safety and regulation, including the use of NMs in cosmetics. The ICCR provides a multilateral framework to maintain global consumer protection, by working towards regulatory convergence.

## 5. Perspectives/Outlook

Nanomaterials can be used in cosmetic industry in various forms and types. It is a fact that due to the progress and development of R&D in the cosmetic industries, an increasingly large number of nanostructures are tested in cosmetics. The future target is clear: to find the safest and most appropriate nanomaterial to use in multiple cosmetic applications but with the lowest cost. Also, it is mandatory to mention that “*EU is to promote the development and validation of alternative methods that adhere to the 3Rs principle, and to provide a level of safety equivalent to that obtained through animal testing while using fewer animals, causing less suffering, or avoiding any use of animals. In view of the ban, the need for implementing non-animal alternatives is particularly crucial for safety assessment of cosmetic ingredients/products because safety data can only be drawn from alternative methods, meaning that the 3Rs choices are effectively restricted to 1R (i.e., Replacement of animal testing). In view of this, the SCCS considers all available scientific data, taking into account the testing and marketing bans in force under Regulation (EC) No 1223/2009*”. The latter statements are the first major target to follow, because the variety of nanomaterials in cosmetic is tremendous, but the ethics must be kept intact.

The future will show what the major future trends and needs in the nanocosmetics sections will be [85]. Sustained and controlled release of sunscreens, with improved moisturizing capacity along with anti-aging properties could potentially be established. Some very promising delivery systems are being investigated, with an array of practical applications. While some may not find their way out of the laboratories, others have the potential to bring major breakthroughs in the cosmetic world. An exceptionally unique nanomaterial, carbon nanobuds have been identified with the combined properties of carbon nanotubes and fullerenes. They are prepared by combining the two most common allotropes of carbon, fullerenes, and carbon nanotubes. Carbon nanotubes are specifically covalently bounded to fullerene-like “sprouts/buds”. They possess remarkably good field emitting properties. This may be of particular use in the manufacture of lipsticks and mascaras. Furthermore, new nanosized metal pigments, in addition to the widely known titanium dioxide and zinc oxide, should be continuously investigated and proposed for cosmetics. In addition, specifically shaped nanoparticles made to fill the uneven surfaces—especially after plastic surgery—for aesthetic appearance of body parts, may open new horizons. Another promising area could be the triggered release of nanomaterials on the skin facilitated by skin pH gradient. Enzymes conjugated with protein nano-carriers have also been receiving attention, due to their exceptional water binding capacity into the stratum corneum. The number of patent applications registered in the last five years suggests that there could be an increased activity in the protein conjugation area in the immediate years ahead. Despite the plethora of benefits nanocosmetics may bring one cannot deny the potential dangers that are linked to some nanomaterials. Furthermore, risk estimation of nanomaterials ought to be carried out on an item-by-item basis, using relevant information.

If the presented cosmetic nanomaterial development is allowed to bloom after the currently ongoing nanotoxicity and safety research incentives have had a chance to affect regulations and guidelines for production and use of nanomaterials, and if the necessary commercial support is in place to carry the materials presented in recently published scientific articles and granted patents to the market, we can soon expect to see a range of new nanotechnology-based cosmetic products on the shelves of the nearest drug store [2]. These include new sunscreens containing diamond nanoparticles attenuating UV radiation while simultaneously removing the free radicals generated by UV light, long-lasting hair dyes with carbon nanotubes providing smoothening, volumizing, and anti-damaging effects, dental-care products containing fluoride nanoparticles treating dentin sensitivity by precluding the transmission of pain signals to the brain as well as alumina and rod-shaped hydroxyapatite nanoparticles providing efficient polishing while simultaneously inducing long-lasting tooth remineralization, and anti-age creams with active ingredients embedded in nanoparticles of synthetic polymers acting as skin permeability enhancers [2]. The major conclusion is undoubtedly the following: “*The cost is not the only important issue, safety and the application of alternative testing methods for toxicity are of crucial importance as well*”.

## Figures and Tables

**Table 1:** Catalogue of cosmetic ingredients registered in EU.

EC/List Name	EC	CAS	Type	Name
Carbon black	215-609-9	1333-86-4	Colorant	CI 77266/Carbon black
Titanium dioxide	236-675-5	13463-67-7	Colorant/UV filter	CI 77891/TiO_2_
Zinc oxide	215-222-5	1314-13-2	Colorant/UV filter	CI 77947/ZnO
2,2’-methylenebis(6-(2H-benzotriazol-2-yl)-4-(1,1,3,3-tetramethylbutyl)phenol)	403-800-1	103597-45-1	UV filter	Methylene bis-benzotriazolyl tetramethylbutylphenol
1,3,5-Triazine, 2,4,6-tris([1,1’-biphenyl]-4-yl)	479-950-7	31274-51-8	UV filter	Tris-biphenyl triazine
Al_2_O_3_	215-691-6	1344-28-1	Other functions	Alumina
Cu	231-159-6	7440-50-8	Other functions	Colloidal copper
Au	231-165-9	7440-57-5	Other functions	Colloidal gold
Pt	231-116-1	7440-06-4	Other functions	Colloidal platinum
Ag	231-131-3	7440-22-4	Other functions	Colloidal silver
Cu	231-159-6	7440-50-8	Other functions	Copper
(C60-Ih)[5,6]fullerene	99685-96-8	99685-96-8	Other functions	Fullerenes
Fullerene C_70_	634-223-5	115383-22-7	Other functions	Fullerenes
Fullerene, multiwalled	923-072-3	N/A	Other functions	Fullerenes
Fullerenes C_60_/C_70_	682-073-4	131159-39-2	Other functions	Fullerenes
Fullerenes C_60_/C_70_	943-307-3	131159-39-2	Other functions	Fullerenes
Au	231-165-9	7440-57-5	Other functions	Gold
silicon dioxide; synthetic amorphous silicon dioxide (nano)	231-545-4	7631-86-9	Other functions	Hydrated silica
Hydroxylapatite (Ca_5_(OH)(PO_4_)_3_)	215-145-7	1306-06-5	Other functions	Hydroxyapatite
Pentacalcium hydroxide tris(orthophosphate)	235-330-6	12167-74-7	Other functions	Hydroxyapatite
Silicic acid, lithium magnesium sodium salt	258-476-2	53320-86-8	Other functions	Lithium magnesium sodium silicate
Platinum	231-116-1	7440-06-4	Other functions	Platinum
silicon dioxide; synthetic amorphous silicon dioxide (nano)	231-545-4	7631-86-9	Other functions	Silica
Amorphous silica	614-122-2	67762-90-7	Other functions	Silica dimethicone silylate
Silane, dichlorodimethyl-, reaction products with silica	271-893-4	68611-44-9	Other functions	Silica dimethyl silylate
Silanamine, 1,1,1-trimethyl-*N*-(trimethylsilyl)-, hydrolysis products with silica	272-697-1	68909-20-6	Other functions	Silica silylate
Ag	231-131-3	7440-22-4	Other functions	Silver
Silicate(2-), hexafluoro-, disodium, reaction products with lithium magnesium sodium silicate	285-349-9	85085-18-3	Other functions	Sodium magnesium fluorosilicate
No entry in European Chemicals Agency chemicals database	N/A	N/A	Other functions	Sodium magnesium silicate
No entry in ECHA’s chemicals database	N/A	N/A	Other functions	Sodium propoxyhydroxypropyl thiosulfate silica
2-Propenoic acid, 2-methyl-, polymer with ethenylbenzene	618-461-7	9010-92-8	Other functions	Styrene/acrylates copolymer
